# Haplotype Mapping of a Diploid Non-Meiotic Organism Using Existing and Induced Aneuploidies

**DOI:** 10.1371/journal.pgen.0040001

**Published:** 2008-01-04

**Authors:** Melanie Legrand, Anja Forche, Anna Selmecki, Christine Chan, David T Kirkpatrick, Judith Berman

**Affiliations:** Department of Genetics, Cell Biology and Development, University of Minnesota, Minneapolis, Minnesota, United States of America; Washington University, United States of America

## Abstract

Haplotype maps (HapMaps) reveal underlying sequence variation and facilitate the study of recombination and genetic diversity. In general, HapMaps are produced by analysis of Single-Nucleotide Polymorphism (SNP) segregation in large numbers of meiotic progeny. Candida albicans, the most common human fungal pathogen, is an obligate diploid that does not appear to undergo meiosis. Thus, standard methods for haplotype mapping cannot be used. We exploited naturally occurring aneuploid strains to determine the haplotypes of the eight chromosome pairs in the C. albicans laboratory strain SC5314 and in a clinical isolate. Comparison of the maps revealed that the clinical strain had undergone a significant amount of genome rearrangement, consisting primarily of crossover or gene conversion recombination events. SNP map haplotyping revealed that insertion and activation of the *UAU1* cassette in essential and non-essential genes can result in whole chromosome aneuploidy. *UAU1* is often used to construct homozygous deletions of targeted genes in C. albicans; the exact mechanism (trisomy followed by chromosome loss versus gene conversion) has not been determined. *UAU1* insertion into the essential *ORC1* gene resulted in a large proportion of trisomic strains, while gene conversion events predominated when *UAU1* was inserted into the non-essential *LRO1* gene. Therefore, induced aneuploidies can be used to generate HapMaps, which are essential for analyzing genome alterations and mitotic recombination events in this clonal organism.

## Author Summary

Candida albicans, a heterozygous diploid yeast, is the most prevalent fungal pathogen. It often acquires resistance to anti-fungal drugs via genome-altering recombination events. In many organisms, recombination events are analyzed using Haplotype Maps (HapMaps), which show the location of different alleles on each chromosomal homolog. Conventional HapMaps are constructed by following allelic markers as they segregate in meiotic progeny. Because C. albicans has not been shown to undergo meiosis, construction of a Candida HapMap has not been possible. We exploited the presence of whole chromosome aneuploidies in mitotic progeny of C. albicans to detect skewed ratios of different alleles, thereby determining the relationships between these alleles on each chromosomal homolog. This facilitated the construction of a HapMap for the most commonly used C. albicans laboratory strain. We then used this HapMap to identify all of the recombination events in a clinical isolate relative to the laboratory reference strain. Finally, we used this mapping approach to investigate the molecular mechanisms that affect the C. albicans genome when it is subjected to a common gene disruption technique. Our rapid HapMap construction method is generally applicable to any organism for which whole-chromosome aneuploidy events can be identified.

## Introduction

Researchers are using Haplotype Maps (HapMaps) with increasing frequency, due to their utility in revealing underlying sequence variation [1]. HapMaps facilitate the study of factors influencing recombination and genetic diversity, as the three primary activities that alter haplotype structure are mutation, recombination, and selection [2,3]. The haplotype is the map of the location of specific gene alleles on each specific chromosome homolog. If an organism is haploid, containing only one copy (homolog) of each chromosome, the haplotype is the genetic sequence of that chromosome. In diploid organisms, alleles of two different genes that reside on the same chromosome can be located on the same homolog (in *cis*) or on different homologs (in *trans*).

In most organisms, HapMaps are produced by analysis of Single-Nucleotide Polymorphism (SNP) segregation in large numbers of meiotic progeny. Candida albicans, the most common human fungal pathogen, is an obligate diploid organism that is not currently known to undergo meiosis and, unlike its close relative Saccharomyces cerevisiae, no haploid or homozygous form of C. albicans is available. As a result, the haplotypes of each chromosome have yet to be determined; researchers usually do not know if two heterozygous mutations, in genes that are found on the same chromosome, occur on the same homolog or on different homologs. As a consequence, linkage mapping or recombinational analysis is not possible in C. albicans using standard methods. However, investigation of recombination is vitally important, as a large proportion of events leading to antifungal drug resistance in clinical settings may involve recombinational mechanisms [4].

Sequencing of the C. albicans genome revealed a high level of natural heterozygosity (55,655 SNPs for the entire 32Mb diploid genome) [5]. Additional studies showed that differences in SNPs are associated with different phenotypes [6]. Two newly-developed microarray technologies – whole genome single-nucleotide polymorphism (SNP) analysis, and comparative genome hybridization (CGH) analysis, permit the analysis of specific genotypes and of the number of copies of each allele, respectively. SNP microarrays are composed of DNA sequences known to be polymorphic in the genome; both alleles are present on the array, and hybridization of DNA from the strain of interest to one or both of the allele sequences indicates the homozygosity or heterozygosity at that position in that strain [7]. SNP arrays can detect loss of heterozygosity (LOH) but do not distinguish between alleles that are present in *cis* or in *trans* and thus cannot detect reciprocal cross-over events. In C. albicans, cross-overs between highly repetitive Major Repeat Sequence (MRS) regions, which are found on essentially all chromosomes, are thought to have resulted in multiple translocation events found in divergent clinical strains [8].

CGH microarrays are composed of an array of DNA sequences from every ORF in the genome, and hybridization of DNA from the strain of interest determines the copy number of each ORF relative to the copy number of the corresponding ORF in the diploid reference strain [9]. When the CGH intensity data is plotted as a function of position on the genetic map, chromosomes or chromosomal segments that exhibit a deviation in copy number (aneuploidy) are readily identified. The combined use of both SNP and CGH microarrays can identify heterozygous DNA sequence polymorphisms (which will be present in a 1:1 ratio in a diploid cell) and demonstrate the copy number of the chromosomes bearing the SNPs.

C. albicans tolerates aneuploidy of all of its chromosomes [8–10]. For example, growth on sorbose induces chromosome 5 (Ch5) monosomy, and duplication of the remaining Ch5 homolog occurs when the monosomic strain is grown on rich media [11]. Similarly, chromosome loss followed by duplication of the remaining homolog is one mechanism for spontaneous *MTL* homozygosis [12]. Natural whole chromosome trisomies have been reported in C. albicans lab strains: Ch1 trisomy and Ch2 trisomy occur in commonly used strains [8,9] and Ch3 trisomy was reported in strains selected for fluconazole resistance [13]. Furthermore, CGH analysis demonstrated that all C. albicans chromosomes can be aneuploid and that approximately 50% of Flu^R^ laboratory and clinical strains contained at least one aneuploidy [4]. It has been hypothesized that changes in chromosome copy number provide a means for genetic variation in C. albicans [14].

We realized that strains that are aneuploid can be exploited to determine the *cis*/*trans* linkage of the SNPs in a non-meiotic heterozygous organism like *C. albicans.* Strains in which a chromosome is homozygous (either due to monosomy or chromosome duplication followed by loss of the heterozygous chromosome) will give SNP ratios of 1:0 or 0:1. Addition of a third copy of the chromosome will change the ratio of the SNPs on that chromosome from 1:1 to 1:2 or 2:1, depending on which allele is present on the extra chromosome, allowing assignment of a particular SNP allele to a specific chromosome.

In C. albicans, the *UAU1* deletion cassette [15] was developed to generate homozygous disruption or deletion alleles using only a single transformation step. The cassette includes 3 segments: a nonfunctional 3′ deletion copy of *URA3*, a functional *ARG4* and a nonfunctional 5′ deletion copy of *URA3* that shares 530bp of sequence identity with the 3′ deletion segment. In the *UAU1* state, the cassette expresses *ARG4* but not *URA3*. Recombination between the 530bp of homologous sequence of the two *ura3* segments produces an intact *URA3* gene and excision of *ARG4*, thus expressing *URA3* but not *ARG4*. Transformation of the *UAU1* cassette into a locus, followed by screening for segregants that express both *ARG4* and *URA3*, results in the isolation of homozygous mutations, although the mechanism by which homozygosis occurs (gene conversion or chromosome duplication followed by chromosome loss) has not been determined. When an essential gene is targeted, the target locus becomes trisomic, carrying copies of *URA3*, *ARG4* and a wild-type copy of the targeted gene. However, the presence and extent of the trisomy (whole-chromosome versus segmental) has not been investigated.

We have developed a novel approach to determine the haplotype of C. albicans strains using SNP and CGH array analysis of strains carrying either naturally-occurring or *UAU1*-induced aneuploidies. If a strain is monosomic (or if it is a disomic homozygote) the ratio of the two SNP alleles on that chromosome will be 1:0 or 0:1. If a strain is carrying an extra copy of a chromosome (trisomy), the ratio of the SNPs on that chromosome will be 1:2 or 2:1, depending on which allele is present on the extra chromosome. These altered ratios enable the assignment of all SNP alleles to a specific chromosomal homolog. We exploited this feature of aneuploid strains to generate SNP haplotype maps of laboratory and clinical C. albicans strains, finding that a significant amount of recombination has occurred in the clinical isolate relative to the laboratory strain. We also investigated the genome changes resulting from the activation of the *UAU1* cassette in essential and non-essential genes located on Ch1, and found that trisomy of the full-length Chromosome 1 carrying the target gene results when an essential gene is targeted, while homozygous mutants are obtained primarily by gene conversion when a nonessential gene is targeted. Our results show that the SNP haplotype map can be used to investigate recombination events across the entire genome.

## Materials and Methods

### Strains and Media

C. albicans was routinely cultured in YEPD (1% Yeast Extract, 2% Peptone, 2% Dextrose, 2% Agar) supplemented with 20mg/L uridine at 30 °C. Selection was done on minimal medium (6.7% yeast nitrogen base plus ammonium sulfate, without amino-acids, 2% dextrose, 2% agar) supplemented with the appropriate amino-acid mix. The strains used to determine SC5314 haplotype maps are derivatives of SC5314 that were shown to be homozygous or trisomic for a specific chromosome when analyzed by CGH and SNP arrays (Table 1). The strains used to determine T118 haplotype maps are drug-resistant derivatives from fluconazole-sensitive clinical isolate T118 [16,17] that were found to be homozygous or trisomic for a specific chromosome when analyzed by CGH and SNP arrays (Table 2). For the *UAU1* analysis, chromosome trisomy was artificially induced in SN76 [18], which is derived from SC5314. The strains constructed for the *UAU1* analysis are presented in Table 3.

**Table 1:**
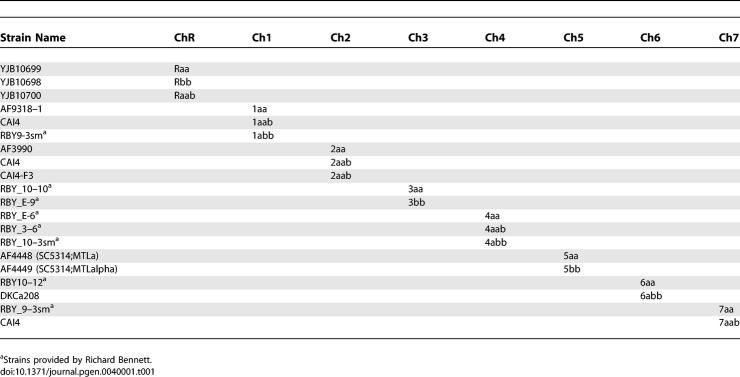
Strains Used To Determine Haplotypes of Strain SC5314

**Table 2:**
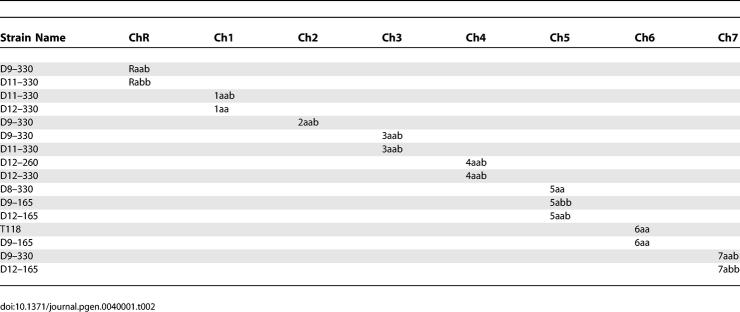
Strains Used To Determine Haplotypes of Strain T118

**Table 3:**
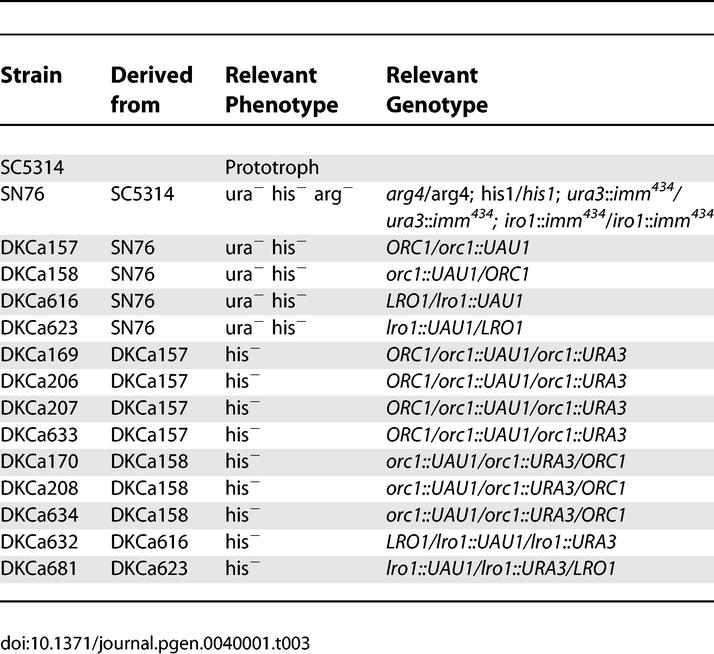
Yeast Strains Constructed for the *UAU1* Analysis

### Transformation in C. albicans

Integration of the *UAU1* cassette into the C. albicans genome was done by homologous recombination using a PCR-based cassette method [19]. Oligonucleotides used in this work are listed in Table 4. The *orc1::UAU1* and *lro1::UAU1* cassettes were obtained by amplifying the *UAU1* cassette from pBME101 [15] with oligonucleotides CaORC1-pBME101-F/R and CaLRO1-pBME101-F/R, respectively. PCR products were concentrated by ethanol precipitation and then transformed into SN76, using a modification of the standard Lithium/Acetate transformation protocol. Overnight cultures were inoculated into 50ml YEPD+uri at an OD_600_ of ∼0.05 and incubated with shaking at 30°c until the OD_600_ reached ∼0.5. Cells were pelleted, washed with 5ml sterile water and resuspended in 500ul of TE/LiOAc (10mM Tris HCl [pH 7.5], 1mM EDTA [pH 8], 0.1M Lithium Acetate). Cells were transferred to a 1.5ml eppendorf tube, pelleted and resuspended in 300 ul of TE/LiOAc. One hundred microliters of this cell suspension was mixed with 5ul of Salmon Sperm Single Stranded DNA (10 mg/ml) and the transforming DNA, and then incubated at room temperature for 30 minutes. 700ul of PLATE mix (10mM Tris HCl [pH 7.5], 1mM EDTA [pH 8], 0.1M Lithium Acetate, 50% polyethylene glycol 3350) was added and mixed by pipetting slowly. The mixture was incubated at room temperature overnight and then incubated at 42 °C for one hour. Cells were pelleted, resuspended in 200 ul of sterile water and plated on appropriate media at 30 °C for 3 days.

**Table 4:**
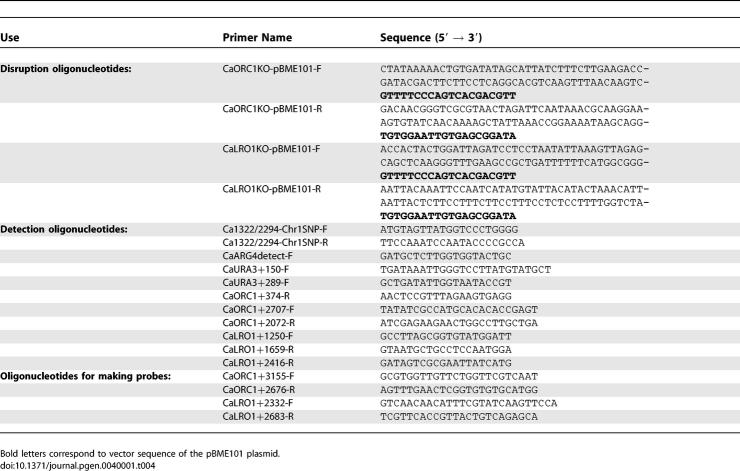
Oligonucleotides Used in This Study

Transformed cells were plated on Min+His+Uri plates to select for Arg^+^ transformants. The *ORC1-UAU1* and *LRO1-UAU1* transformants were screened by colony PCR with oligonucleotides CaARG4detect-F/CaORC1+374-R and Ca1322/2294Chr1-SNP-F/CaURA3+289-F, respectively. The genomic DNA of positive transformants was extracted from a single colony and the genotype of the *ORC1/orc1::UAU1* heterozygotes was confirmed by PCR with oligonucleotides CaORC1+2707-F/CaURA3+289-F and CaARG4detect-F/CaORC1+374-R. Genotypes of the *LRO1/lro1::UAU1* heterozygotes were confirmed by PCR with oligonucleotides Ca1322/2294Chr1-SNP-F/CaURA3+289-F and CaARG4detect-F/CaLRO1+2416-R. The presence of the wild type gene in the heterozygotes was confirmed by PCR with oligonucleotides CaORC1+2707-F/CaORC1+2072-R for *ORC1* and Ca1322/2294Chr1-SNP-F/CaLRO1+1659-R for *LRO1*. The SNP present within the *ORC1* open reading frame was amplified by PCR with oligonucleotides CaARG4detect-F/CaORC1+374-R and sent for sequencing to determine which Ch1 homolog was targeted. DKCa157 is an *ORC1/orc1::UAU1* heterozygote in which the *UAU1* cassette integrated into one of the Ch1 homologs, while DKCa158 is a second heterozygote in which the *UAU1* cassette integrated into the other Ch1 homolog. The SNP located within the *LRO1* open reading frame was amplified by PCR with oligonucleotides Ca1322/2294Chr1-SNP-F/R. The PCR product was then incubated overnight at 37 °C with restriction enzyme *Bcc*I. We selected two *LRO1/lro1::UAU1* strains such that a different homolog was targeted in each strain (DKCa616 and DKCa623).

DKCa157, DKCa158, DKCa616 and DKCa623 cells were patched on YEPD+uri, grown at 30°C for 48h and replica-plated on Min+His to select for Arg^+^ Ura^+^ derivatives. Genomic DNA was extracted from single Arg^+^ Ura^+^ colonies, as described previously. The presence/absence of the wild type *ORC1* and *LRO1* genes was assessed by PCR with oligonucleotides CaORC1+2707-F/CaORC1+2072-R and Ca1322/2294Chr1-SNP-F/CaLRO1+1659-R, respectively. As potential Ch1 trisomy candidates, we selected Arg^+^ Ura^+^ derivatives in which a wild-type copy of the target gene *ORC1* or *LRO1* is still present (DKCa169/DKCa634 for *ORC1* and DKCa632/DKCa681 for *LRO1*). We also selected 10 Arg^+^ Ura^+^ derivatives from DKCa616 and from DKCa623 that lacked the wild type *LRO1* gene, to investigate the mechanism by which homozygosis occurs for non-essential genes disrupted by the *UAU1* cassette.

### Southern Analysis

To confirm trisomy of the target locus, Southern blot analysis was performed as described previously [9]. Genomic DNAs from *orc1::UAU1* and *lro1::UAU1* Arg^+^ Ura^+^ derivatives were digested with *Mfe*I and *Xba*I + *Bbs*I respectively. DNA probes were generated by PCR using the DIG DNA Labeling Kit (Roche) according to the manufacturer's instructions. The *ORC1* probe was generated with the oligonucleotides CaORC1+3155-F/CaORC1+2676-R and the *LRO1* probe was generated with the oligonucleotides CaLRO1+2332-F/CaLRO1+2683-R. The size of each fragment was determined based on the genome sequence.

### CGH Array and SNP Array

CGH and SNP arrays were performed as described previously [7,9] and were plotted without data smoothing [4]. Out of 150 defined SNPs [20], 122 SNPs were used in this study. Most of those omitted were homozygous in SC5314 or were part of one large SNP marker - only one SNP for each of this type of locus is presented on the map. The order of SNP alleles presented along each chromosome is based on the physical map and the published sequence of the genome [20], as it cannot be determined directly from SNP haplotype mapping.

## Results/Discussion

### Haplotype Mapping of SC5314 Using LOH and/or Aneuploidy

**Figure 1:**
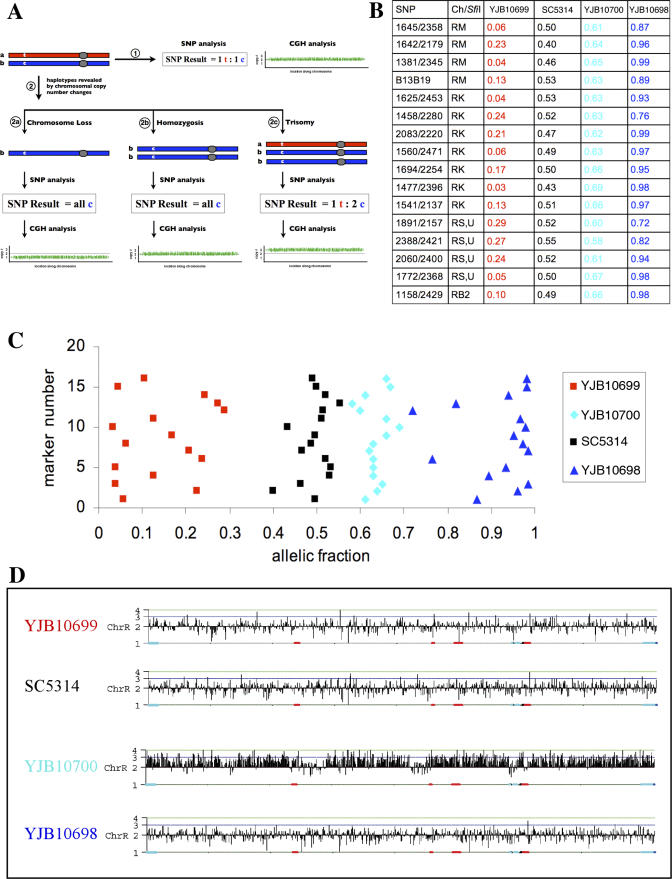
Allelic Fractions from Aneuploid Strains Can Be Used To Generate SNP Haplotype Maps (A) Haplotype determination by chromosomal variation analysis. A typical chromosome pair in C. albicans is depicted in the upper left. One homolog is shown in red, the other in blue, with centromeres indicated as a grey circle. This chromosome pair bears a single nucleotide polymorphism (SNP) on the left arm, shown as a “t” on the red homolog and a “c” on the blue. If this chromosome pair is analyzed (arrow 1), SNP analysis will demonstrate that the two SNP variants are present in a 1:1 ratio, while comparative genome hybridization (CGH) will demonstrate that there are two copies of the entire chromosome. This chromosome pair undergoes an alteration in copy number (arrow 2): simple chromosome loss (2a), loss and homozygosis of the remaining homolog (2b), and duplication of an extra copy of one of the homologs, leading to trisomy (2c). SNP analysis of the chromosome loss event (2a) will show that only one of the two alleles is present, while CGH analysis will indicate a reduction in chromosome number. Analysis of the homozygosis (2b) will give the same SNP result but indicate a diploid chromosome number, while analysis of the trisomy (2c) will show a 1:2 ratio of the SNP alleles and the gain of a third chromosome. These results allow the unambiguous mapping of a particular SNP allele to a particular homolog (designated “a” or “b”). (B) Allelic fractions can be indicative of heterozygous trisomies of whole chromosomes. Allelic fractions (AF) of SNPs on ChR for strains YJB10699, SC5314, YJB10700, and YJB10698, which served as reference strains for the individual genotypes. Allelic fractions were calculated: AF-allele b + AF-allele a/(AF-allele b + AF-allele a). Ranges for genotype calls: AF<0.4−homozygous allele a; AF>0.4 and <0.6 − heterozygous a/b; AF>0.6−homozygous allele b. (C) Plot of allelic fractions for SNP loci on ChR in four reference strains. Allelic fractions for strains YJB10699, SC5314, YJB10700, and YJB10698 (*x*-axis) and the corresponding SNP (*y*-axis). Allelic fractions for strains YJB10699, SC5314, and YJB10698 fall into the expected ranges. For strain YJB10700, the allelic fractions fall between the heterozygous and homozygous range indicating a possible trisomy. (D) CGH profiles of ChR for reference strains YJB10699, SC5314, YJB10700, and YJB10698. CGH profiles for ChR for the four reference strains confirm the diploid homozygous state for strains YJB10699 (homozygous a/a) and YJB10698 (homozygous b/b), the heterozygous diploid state for strain SC5314 (a/b), and the trisomic heterozygous state for strain YJB10700 (2× allele a, 1× allele b).

Single Nucleotide Polymorphism (SNP) microarrays determine if specific regions of a diploid genome are heterozygous or homozygous [7]. However, SNP arrays alone cannot determine the *cis* or *trans* arrangement of linked heterozygous alleles. To generate a haplotype map of C. albicans, we determined the *cis*/*trans* linkage of SNPs in strains carrying aneuploidies. The altered chromosomal copy number was first detected by Comparative Genome Hybridization (CGH) microarrays [9]. Theoretically, the addition of a third copy of a chromosome will change the ratio of the SNPs on that chromosome from 1:1 to 1:2 or 2:1, while loss of one of the two homologs will result in a 1:0 allele ratio for one of the alleles for each SNP. We exploited these alterations in allelic ratio to assign a particular SNP allele to a specific chromosome (Figure 1A).

To test the feasibility of this SNP haplotype mapping approach, we analyzed three C. albicans yeast strains derived from laboratory strain SC5314 for correlations between changes in chromosomal copy number and SNP ratios. The allelic fractions of the chromosome R (ChR) SNPs in these strains were compared to the allelic fractions in the diploid strain SC5314 (Figure 1B and 1C). As expected, SC5314 alleles were heterozygous for ChR (an allelic fraction of ∼0.5, which is equivalent to a SNP ratio of 1:1) whereas alleles in strains YJB10698 and YJB10699 were generally homozygous (allelic fraction ∼0 or ∼1 (0:1 or 1:0) respectively). Importantly, all SNP alleles on ChR that were 0:1 in strain YJB10698 were 1:0 in YJB10699, and vice versa. We used these results to define the set of ChR SNP alleles in YJB10698 as allele “a” and the set of SNP alleles in YJB10699 as allele “b”. Interestingly, in the third strain, YJB10700, most SNPs had an allelic fraction of ∼0.66 (2:1) for ChR (Figure 1B and 1C), suggesting that the strain is trisomic for ChR. A CGH microarray confirmed the YJB10700 ChR trisomy (Figure 1D). This result, combined with the SNP data, indicates that YJB10700 has two copies of SNP allele set “a” (the ChRa homolog) and only one copy of SNP allele set “b” (the ChRb homolog). Thus, allelic fractions from strains that are aneuploid for a specific chromosome can be used to generate SNP haplotype maps of that chromosome.

We next used aneuploidies to determine the SNP haplotype for all of the chromosomes in SC5314, the most common C. albicans laboratory strain (Tables S1 and S2). SC5314 is the wild-type strain that was sequenced; it is the progenitor of CAI4 and BWP17, the major strains used in laboratory studies. Previous analysis of a large set of strains identified twenty SC5314 derivatives in which at least one of the eight chromosomes was either homozygous or trisomic (Table 1). For example, we included *MTLa/a* and *MTLα/α* derivatives from the *MTLa/α* SC5314 that had been selected previously for their ability to grow on sorbose [7]. Because these two isolates are homozygous for Chr5, SNP array analysis defined haplotype maps for each Chr5 homolog.

**Figure 2:**
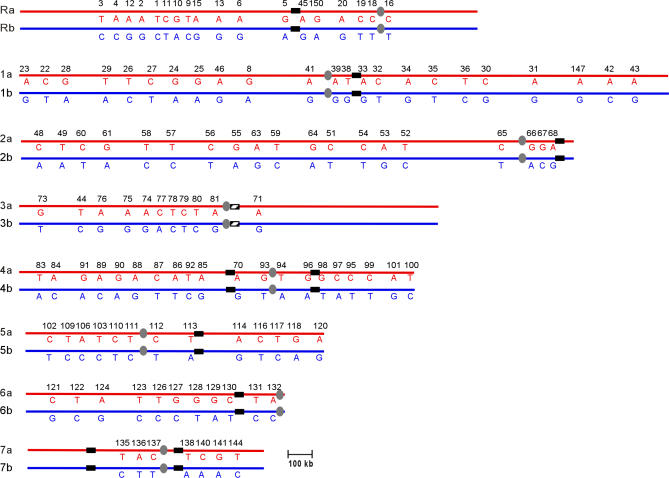
SNP Haplotype Map of Strain SC5314 Chromosomes are listed from ChR, Ch1 through Ch7. Homologs are designated as “a” and “b”. Alleles on homolog “a” are indicated in red, while alleles on “b” are indicated in blue. Horizontal numbers are the unique IDs for each SNP locus. Each vertical pair of capital letters indicates the heterozygous nucleotide present at each SNP. Centromeres are indicated as gray ovals. MRS (or RB2 in the case of Ch3) sequences are indicated as black vertical bars on each chromosome.

The data from multiple aneuploid strains were combined to construct a complete SNP haplotype map of strain SC5314 (Figure 2; Table S1). The current C. albicans SNP map contains 150 SNP markers comprising 561 SNPs and 9 insertions-deletions. On average the map has 1 SNP marker every 111kb across the 16Mb genome [20]. The SNP haplotype map determined for SC5314 in this paper will serve as a reference for subsequent C. albicans haplotype analysis. As was done with the primary DNA sequence [5], we have set the SNP haplotype map of SC5314 as the reference for comparison with other strains. Presentation of the SNP map of SC5314 as un-rearranged is not meant to imply any evolutionary or phylogenetic relationships.

### Recombination Events in a Clinical Isolate Revealed by Haplotype Mapping

One striking feature of the C. albicans genome is its plasticity. Although the genome of laboratory strains grown under standard growth conditions is relatively stable, half of clinical isolates have variant karyotypes [21]. In addition, between 3% and 7% of clinical isolates are naturally homozygous at the *MTL* locus [22,23]. One mechanism leading to loss of heterozygosity (LOH) at *MTL* is homozygosis of the entire chromosome due to a chromosome loss event followed by duplication of the remaining chromosome [12]. Another alternative mechanism for homozygosis requires chromosome duplication followed by loss of the single heterozygous chromosome and retention of the two remaining homozygous chromosomes. In addition, Soll and coworkers have reported conflicting data on the significance of gene conversion at the mating type locus [12,24]. Recent whole-genome CGH array analysis of fluconazole-resistant clinical isolates revealed that approximately 50% of Flu^R^ clinical strains exhibited chromosomal aneuploidies, with up to 20% of the isolates carrying an isochromosomal derivative of Ch5, in which the left arm of the chromosome had been duplicated [4].

In order to gain a better understanding of the genome rearrangements occurring in clinical isolates, we compared the SNP haplotype of SC5314 with the haplotype of a clinically-derived isolate. T118 is a diploid Flu^S^ strain obtained from an HIV-infected patient [16]. Preliminary SNP analysis of T118 detected SNP heterozygosity on Ch2 and Ch4, while at least seven regions (on ChR, Ch1, Ch3, Ch5, Ch6 and Ch7) exhibited LOH in T118. To construct a complete SNP haplotype map, we used isolates previously derived from T118 by serial passaging in the presence of fluconazole [17]. We identified seven individual T118-derived strains that were trisomic or homozygous for one or more chromosomes (Table 2), and used these strains for SNP haplotype analysis (Table S3).

**Figure 3:**
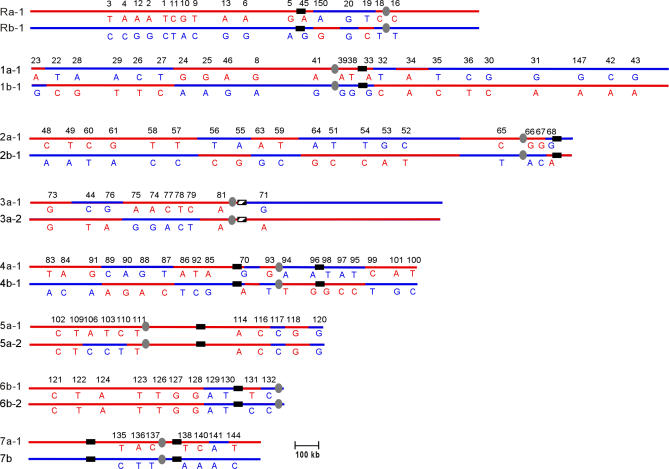
Chromosomal Rearrangements Are Observed in the SNP Haplotype Map of the Clinical Isolate T118 Chromosomes are listed from ChR, Ch1 through Ch7; homolog names were assigned based on the allele type (“a” or “b”) of the SNPs closest to the centromere in reference to the SC5314 haplotype map in Figure 2. If chromosomes were homozygous at the centromere, the two different homologs were numbered −1 and −2 (e.g., Ch3a-1 and Ch3a-2). Alleles on homolog “a” are indicated in red, while alleles on “b” are indicated in blue. Numbers displayed horizontally along the chromosomes are the unique IDs for each SNP locus. Each vertical pair of capital letters indicates the heterozygous nucleotide present at each SNP. Centromeres are indicated as gray ovals. MRS (or RB2 in the case of Ch3) sequences are indicated as black vertical bars on each chromosome. Recombination tracts are identified based on rearrangement of alleles compared to strain SC5314. Some SNPs used in the SC5314 map are not present in the T118 map due to technical issues.

Comparison of the T118 haplotype map (Figure 3) with the SC5314 map showed that the T118 clinical isolate has a number of chromosomal alterations involving every chromosome relative to the SNP map for SC5314. However, these alterations are only those that are detectable by the current SNP array, and so represent only the minimal amount of variation between the two strains. Also, as discussed above, presentation of the T118 data as having undergone recombination or alteration does not imply that SC5314 was the un-recombined precursor to T118; interpretations are presented in this manner to facilitate discussion of the relative differences in the two strains. Finally, note that chromosome homolog assignments (i.e. Ch1a or Ch1b) are given based on the allele identity of the SNP closest to the centromere of each chromosome, and that the exact location of each reciprocal exchange event is unknown, although the crossover sites are shown as occurring half-way between the flanking SNPs in Figure 3.

Chromosomes 2, 4 and 7 exhibited very simple alterations in T118. Ch2 had five crossovers relative to SC5314, while Ch4 had six. Ch7 exhibited a single gene conversion (GC) event, at SNP 141. Chromosomes R, 1 and 3 had slightly more complex alterations, consisting of both crossovers and GCs. On ChR, there were two crossovers, between SNPs 45 and 150 and between 19 and 18, with a region of homozygosity between these flanking crossovers. Ch1 had two simple crossover events on the left arm of the chromosome. On the right arm, a third crossover between SNPs 32 and 33 was flanked by a GC event at SNP 34; this alteration could be an independent GC or it could have occurred at the same time as the adjacent crossover. Ch3 had two GC events, one near the left telomere (SNP 73) and the other to the left of the centromere (SNP 81). A crossover occurred between the two GC events. Because it is the most telomere-proximal SNP marker assayed, the homozygosity observed at SNP 73 could be the result of a standard gene conversion event, a reciprocal crossover followed by chromosome mis-segregation, or break-induced replication (BIR).

Chromosomes 5 and 6 exhibited a complex pattern of rearrangements. In strain T118, Ch5 was completely homozygous for homolog 5a except for two regions of sequence deviation; SNPs 117 to 120 on the right arm of Ch5 and SNPs 106 to 110 on the left arm of the chromosome (Figure 3). SNPs 117 and 120 are homozygous for the Ch5b allele, rather than the Ch5a alleles present elsewhere. This change must have occurred prior to the events that led to the homozygosis of Ch5, and resulted either from multiple crossover events centromere proximal to both 117 and 120 and on either side of SNP 118, or from linked crossover and GC events. Subsequent formation of the tract of internal heterozygosity at SNPs 106 to 110 was more complicated. Either initial crossovers occurred between SNPs 110 and 111 and between SNPs 106 and 109, or the entire region was involved in a gene conversion event. This would have been followed directly by a chromosome mis-segregation event in which the two Ch5a sister chromatids segregated together, resulting in homozygosis of the majority of Ch5 sequences and leaving only a small heterozygous tract on the left arm of the chromosome.

Ch6 was completely homozygous in T118 except for SNP 131. This homozygosis likely resulted from a failure to segregate the Ch6b sister chromatids followed by loss of the heterozygous Ch6a homolog; chromosome loss followed by duplication of the remaining homolog would not have accounted for the heterozygosity at SNP 131. Comparison of Ch6 SNP maps in T118 and SC5314 indicates that the chromosome mis-segregation event leading to Ch6 homozygosis must have been preceded by a crossover event between SNPs 128 and 129 and a gene conversion event at SNP 131.

While some of the chromosomal recombination events occurred in or adjacent to the Major Repeat Sequence (MRS) tracts (on Ch3, Ch4 and Ch6), the majority of the changes (27/30 events) were not associated with an MRS. Thus, the MRS appears to be a site of recombination, albeit not one that is highly predominant. An expansion of this type of haplotype analysis using further clinical isolates will allow us to identify recombination events that may be linked to the acquisition of antifungal drug resistance in Candida albicans.

### Artificial Induction of Trisomy Allows Haplotype Mapping

The SNP haplotype mapping method described above requires aneuploid strains. To make this approach more widely applicable, a method for inducing trisomy of any desired chromosome is needed. One potential method is the use of the *UAU1* deletion cassette [15], which was designed to construct homozygous C. albicans deletion strains. Previous studies have shown that when the *UAU1* cassette is inserted into an essential gene and Ura^+^ Arg^+^ selection is applied, strains that arise have three copies of the locus of interest. However, the organization of the extra copy has not been explored. We used the *UAU1* cassette to ask if triplication arises by whole chromosome or by segmental trisomy.

We used the *UAU1* cassette to target an essential gene as well as a non-essential gene. Both genes were on Ch1 and both contained SNPs that allowed us to follow the fates of the individual homologs. We selected orf19.3000, *CaORC1*, a homolog of the essential *S. cerevisiae ORC1* gene, which encodes the largest subunit of the origin recognition complex. Because of its central role in DNA replication initiation, we assumed that the C. albicans Orc1p also would be essential for cell viability. A SNP present in the *ORC1* open reading frame allowed us to distinguish between the alleles on each Ch1 homolog.

**Figure 4:**
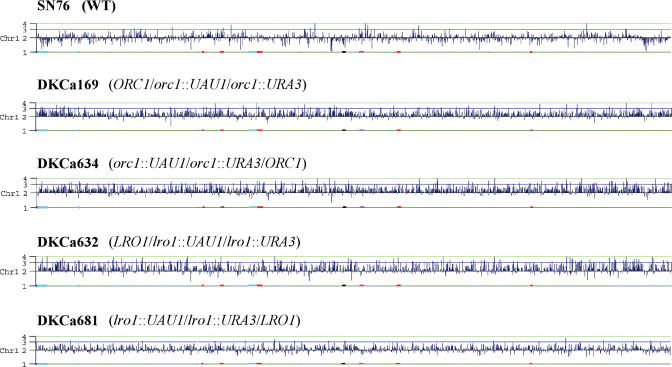
Chromosome Map Display of CGH Data on Ch1 for Arg^+^ Ura^+^ Derivatives and the Parental Strain A view of the extent of copy number changes plotted across the complete length of Ch1 using chromosome map [9]. CGH data were ordered along the chromosome as described in Materials and Methods. Data were plotted as log2 values, which correspond to the chromosome copy number indicated on the *y*-axis.

We deleted one copy of *CaORC1* by integrating the *UAU1* cassette into the *ORC1* locus in parental strain SN76 [18], an SC5314 derivative. Colony PCR identified two correct transformants out of 24 transformants screened. Sequencing of the SNP in the two heterozygotes indicated that the *UAU1* cassette integrated into one *ORC1* allele in the first transformant (DKCa157) and into the other *ORC1* allele in the second transformant (DKCa158). Single colonies from both heterozygotes were grown under selection for Arg^+^ Ura^+^ derivatives, which were analyzed by PCR to detect the presence of the *UAU1* cassette (Arg^+^), the *URA3* recombinant cassette (Ura^+^) and the wild-type *ORC1* allele (see Materials and Methods). One hundred percent of the Arg^+^ Ura^+^ derivatives maintained a wild type *ORC1* allele, providing strong support for the idea that CaOrc1p is essential for cell viability in C. albicans. To assess the extent of the trisomy in these strains, we performed SNP microarray analysis on seven Arg^+^ Ura^+^ derivatives (three from DKCa157 and four from DKCa158) that by PCR and Southern analysis were trisomic at the *ORC1* locus. Trisomy of the full-length Ch1 was observed in three of the seven strains (43%) (DKCa157-derived DKCa169 and DKCa633 and DKCa158-derived DKCa634) (Figure 4), which allowed us to determine the SNP haplotypes of Ch1 in those isolates. In the four remaining Arg^+^ Ura^+^ derivatives (57%), we observed a 1:1 SNP ratio, although we did detect unselected aneuploidies including trisomy of other chromosomes (e.g., Ch2 and Ch7 in DKCa170). Further analysis of these strains indicated that they possessed four copies of the *ORC1* locus – two wild-type, one bearing the *UAU1* cassette and the other bearing the recombined *UAU1* Ura^+^ derivative. These data explain the 1:1 Ch1 SNP ratio, and may indicate that *ORC1* exhibits a mild haplo-insufficiency in Candida albicans, resulting in selective pressure to increase the copy number of the wild-type *ORC1* gene when one copy is deleted.

In all strains, including the parental strain, we observed a partial loss of heterozygosity on Ch2, which is also observed in RM1000, an ancestor of SN76. This change occurred in the process of constructing RM1000 and therefore is expected to be present in all descendants. We also observed trisomy of Ch6 in parental strain SN76 as well as in all of its derivatives. This change is specific to the SN76 isolate used in this study, since CGH analysis of an independent SN76 isolate did not detect Ch6 trisomy (data not shown).

We next asked about the fate of non-essential genes disrupted with the *UAU1* cassette. Previous reports have documented the presence of three copies of genes (allelic triplications) as a result of *UAU1* activation in a non-essential gene [15]. We disrupted *LRO1* (orf19.6018), which is also on Ch1 and includes a SNP (1322/2294). The homolog in S. cerevisiae, Lro1p, is an acyltransferase whose function is not essential for viability in S. cerevisiae. We integrated the *UAU1* cassette into the *LRO1* locus and screened 24 transformants by colony PCR, obtaining 10 transformants that had correctly integrated into the *LRO1* locus. Two heterozygotes (DKCa616 and DKCa623), representing an insertion in each of the two *LRO1* alleles, were selected after SNP typing.

Singles colonies from both heterozygotes were grown on SDC-arg-ura to select for Arg^+^ Ura^+^ derivatives in which the *UAU1* cassette has been activated. The majority of the Arg^+^ Ura^+^ derivatives were homozygous diploid deletion strains in which the wild-type *LRO1* sequence was no longer present, consistent with the idea that *LRO1* is non-essential in C. albicans. The frequency of Arg^+^ Ura^+^ cells that maintained the wild-type *LRO1* gene was low, as determined by colony PCR (3% for DKCa616 and 25% for DKCa623). Two of these Arg^+^ Ura^+^ derivatives, one from each heterozygote, were analyzed by Southern-blot and more detailed PCR analysis. The results confirmed that they each contained three different *LRO1* loci (*LRO1, lro1::UAU1* and *lro1::URA3)* (data not shown). CGH microarray analysis detected trisomy of all of Ch1 for one of the two isolates (DKCa632) (Figure 4). The other isolate (DKCa681), appeared to have approximately 2.5 copies of Ch1, a result that is typical of an intermediate ploidy state between disomy and trisomy (Figure 4), suggesting that a copy of the chromosome is being lost (or gained). Thus, activation of the *UAU1* cassette within both essential and non-essential genes can result in whole chromosome trisomies. A major difference between the essential and non-essential *UAU1* insertion results was the frequency (100% for an essential gene, of which 42% exhibited whole-chromosome trisomy; 3%–25% for a non-essential gene, all of which exhibited whole-chromosome trisomy) at which trisomic strains are observed among the Arg^+^ Ura^+^ derivatives. When essential genes are disrupted, selection for the essential gene and for the *ARG4* and *URA3* genes maintains all three copies; for the non-essential gene there is less selective pressure to maintain the copy that carries the wild-type gene.

To date, no study has addressed the precise mechanism by which homozygous insertion mutations are generated following transformation of the *UAU1* cassette. The construction of homozygous mutants with the *UAU1* cassette could be the result of a gene conversion or a chromosome duplication followed by chromosome loss. A consequence of the latter would be LOH of Ch1. We isolated ten Arg^+^ Ura^+^ derivatives from DKCa616 and DKCa623 for which PCR showed that the wild-type *LRO1* sequence was absent, indicating that these isolates are likely to be disomic for Ch1. We analyzed SNPs flanking the *LRO1* gene (1799/2450, centromere-distal to the *LRO1* locus and located within an *Alu*I restriction site, and SNP 1449/2362, centromere-proximal to *LRO1* and located within a *Fok*I restriction site). Both SNPs are located approximately 128kb away from the *LRO1* gene. Most (18/20) isolates remained heterozygous for both of these SNPs. One isolate remained heterozygous for SNP 1449/2362 but became homozygous for SNP 1799/2450, while the last isolate became homozygous for both flanking SNPs. Thus, when the Ch1 gene that is targeted is not essential, the activation of the *UAU1* cassette primarily occurs by gene conversion, in which a relatively short region flanking the target sequence becomes homozygous. This is in contrast to the insertion of the *UAU1* cassette within an essential gene on Ch1; in this case, Arg^+^ Ura^+^ derivatives can arise through whole chromosome duplication, leading to trisomy.

In conclusion, we used SNP and CGH microarray analysis of strains bearing naturally-occurring or induced aneuploidies to elucidate the SNP haplotype map for all eight chromosomes of the Candida albicans reference strain SC5314. Similar analysis of the genome of a clinical isolate, T118, revealed multiple regions of LOH and rearrangements on all eight chromosomes. This implies that crossover and gene conversion recombination events led to the different haplotypes in these two strains. Further, we used SNP haplotype analysis to determine the molecular mechanisms that generate homozygous gene deletions in strains bearing the *UAU1* deletion cassette on Ch1 of Candida albicans. In theory, any organism should be amenable to this type of analysis, if trisomies can be induced or identified in the population. This approach to SNP haplotype mapping promises to greatly increase our understanding of the genome rearrangement and recombination events that occur in C. albicans, especially during the development of antifungal drug resistance.

## Supporting Information

Table S1 Raw Data (Allelic Fractions) Used to Generate the Haplotype for Strain SC5314 (47 KB XLS)

Table S2 Genomic Position of SNPs Used in the SNP Array (2.3 MB XLS)

Table S3 Allelic Fractions for Clinical Isolates (45 KB XLS)
